# Subtype Specific Nasopharyngeal Carcinoma Incidence and Survival Trends: Differences between Endemic and Non-Endemic Populations

**DOI:** 10.31557/APJCP.2020.21.11.3291

**Published:** 2020-11

**Authors:** Ilona Argirion, Katie R Zarins, Krittika Suwanrungruang, Donsuk Pongnikorn, Imjai Chitapanarux, Hutcha Sriplung, Patravoot Vatanasapt, Laura S. Rozek

**Affiliations:** 1 *Department of Environmental Health Sciences, University of Michigan School of Public Health, Ann Arbor, MI, Thailand. *; 2 *Cancer Unit, Srinagarind Hospital, Khon Kaen University, Khon Kaen, Thailand. *; 3 *Lampang Cancer Hospital, Lampang, Thailand. *; 4 *aculty of Medicine, Chiang Mai University, Chiang Mai, Thailand. *; 5 *Songkhla Cancer Registry, Prince of Songkla University, Songkhla, Thailand. *; 6 *Department of Otorhinolaryngology, Faculty of Medicine, Khon Kaen University, Thailand. *

**Keywords:** Nasopharyngeal carcinoma, incidence rates, survival, global epidemiology

## Abstract

**Background:**

While nasopharyngeal carcinoma (NPC) is rare in non-endemic regions such as the North America, endemic countries, such as Thailand, continue to struggle with high incidence and mortality rates. NPC has a complex etiology that varies by histological subtype.

**Methods:**

NPC cases (1990-2014) were identified using the International Classification of Diseases for Oncology (ICD-O) code C11 from the Chiang Mai, Khon Kaen, Lampang, and Songkhla cancer registries and compared to Asian/Pacific Islanders (A/PI) from the US SEER program. Age-standardized incidence rates and changes in annual percent change (APC) for overall and subtype specific NPC were assessed using R and Joinpoint. Kaplan Meier curves were generated in SAS to evaluate differences in survival by sex, year of diagnosis and histological subtype. Five-year relative survival estimates were calculated between 2000-2014.

**Results:**

Non-keratinizing NPC predominated across all registries except Songkhla, where the keretinizing subtype made up ~60% of all reported cases. Incidence of keratinizing NPC significantly decreased among Chiang Mai males between 1996 and 2014 (APC:-13.0 [95%CI:-16.2, -9.6]), Songkhla females (APC:-4.0 [95%CI: -7.4, -0.5]) and males between 2006 and 2014 (APC:-15.5 [95%CI:-25.0, -4.7]), as well as A/PI females (APC:-5.1 [95%CI:-6,7, -3.4]) and males (APC: -4.8 [95%CI:-5.9, -3.7]). Non-keratinizing NPC increased among Songkhla males (APC:4.3 [95%CI:1.8, 6.9]). The keratinizing subtype exhibited the worst survival, while the non-keratinizing undifferentiated subtype had the best survival. Although US A/PI had the highest 5-year relative survival estimates, among the Thai registries Chiang Mai had the best and Lampang the worst survival.

**Conclusion:**

Although US A/PIs exhibited similar rates of NPC as seen in the endemic Thai population, improved tobacco control has led to a decrease in keratinizing NPC incidence irrespective of geography. Additionally, while challenges associate with access to care may still exist among rural Thais, chemoradiation was shown to confer a survival benefit in non-keratinizing NPC treatment.

## Introduction

Arising in the epithelial lining of the nasopharynx, nasopharyngeal carcinoma (NPC) is uniquely characterized by its striking racial and geographic variation. In most of the world, including the United Stated, NPC is considered to be a rare malignancy, with age-adjusted incidence among both genders reported to be less than one per 100,000 population (Parkin DM, 2002). Nevertheless, in endemic regions such as southern China, Southeast Asia, the Middle East and North Africa, NPC continues to contribute to a large proportion of the overall cancer burden (Chang and Adami, 2006). The World Health Organization (WHO) classified NPC into three distinct histological subtypes: keratinizing squamous cell carcinoma (WHO type 1), non-keratinizing carcinoma (WHO type 2), which can further be divided into differentiated and undifferentiated, and basaloid squamous cell carcinoma (WHO type 3) (Barnes et al., 2005; Lo et al., 2004). While basaloid squamous cell carcinoma is very rare and sparsely mentioned in the literature, in high-incidence countries, non-keratinizing squamous cell carcinoma is considered to be the predominant histological subtype, accounting for an estimated 95% of cases (Chang and Adami, 2006; Yu MC, 1996; Zong et al., 1983). Interestingly, keratinizing squamous cell carcinoma comprises the vast majority of NPCs in non-endemic regions, believed to be associated with alcohol and tobacco consumption patterns (Vaughan et al., 1996).

Several epidemiological studies have established the WHO histological classifications as independent prognostic factors for NPC survival (Burt et al., 1992; Levine et al., 1980), non-keratinizing carcinoma has been shown to have significant survival advantages when compared to keratinizing squamous cell carcinoma, with 5-year survival rates of 51% and 6%, respectively (Reddy et al., 1995). Epstein-Barr virus (EBV) has long been implicated as a causal factor in NPC, as well as several other malignancies (Guidry et al., 2017) and is primarily associated with non-keratinizing carcinoma (Niedobitek et al., 1991; Shi et al., 2002). Albeit being ubiquitous, infecting and persisting latently in over 90% of the world’s population (Rickinson AB, 2001), and with numerous studies finding associations between anti-EBV antibodies, tumor burden and prognosis (Barlow et al., 1979; Chan et al., 2002; de Schryver et al., 1974; de-Vathaire et al., 1988; Gunven et al., 1973; Lin et al., 2001; Lin et al., 2004; Lo et al., 1999; Mathew et al., 1981; Mutirangura et al., 1998; Naegele et al., 1982; Nawroz et al., 1996; Neel et al., 1983; Tamada et al., 1984), there has been no strong evidence to suggest a correlation between the distribution of EBV strains and international patterns of NPC (Chang and Adami, 2006). 

Located in Southeast Asia, Thailand is an endemic nation for NPC. Rapid socioeconomic development in this region during the past few decades has led to a decrease in communicable diseases and an increase in cancer related mortality (Vatanasapt et al., 1995). Using data from four of Thailand’s 16 province- and regional- based registries (Tangjaturonrasme et al., 2017), we compared age standardized NPC incidence and mortality rates across Thailand to those in the Asian/Pacific Islander (A/PI) population within the United States, paying particular attention to variability in histological classifications across these regions. 

## Materials and Methods

*Data Selection and Criteria*

**Figure 1:**
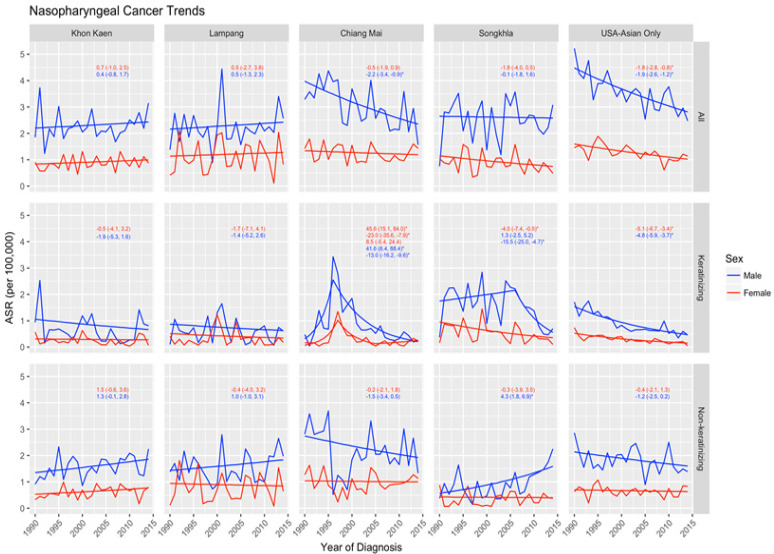
Joinpoint Analyses of Nasopharyngeal Carcinoma by Morphology and Registry (APC [95% CI]). (*) denotes statistical significance at alpha = 0.05

Cancer incidence data between 1990 and 2014 were obtained directly from the Chiang Mai, Khon Kaen, Lampang and Songkhla cancer registries in Thailand (Figure 1) (Alvarez et al., 2018; Demanelis et al., 2015; Mitro et al., 2016; Virani et al., 2014) as well as the Surveillance, Epidemiology, and End Results Program 9 (SEER) (Abazov et al., 2005). For each Thai registry, population estimates by year, age and sex were based on decennial census data from 1990, 2000, and 2010, which were conducted by the Thai National Statistical Office (2002; 2012). Annual intercensal population estimates for the various provinces were calculated using a log-linear function by 5-year sex-specific age groups. Population counts beyond 2010 were modeled by the Office of the National Economic and Social Development Board (Sriplung et al., 2014a; Sriplung et al., 2014b). 

Nasopharyngeal cancer cases were identified in the various registries using the International Classification of Diseases for Oncology (ICD-O) code ‘C11’ and assessed as sex stratified, temporal trends in incidence rates aggregated over 5-year age groups (0-85+). All malignant nasopharyngeal cancer cases identified between the years of 1990 and 2014 were used for these analyses. In addition to cancer site, case information included age, sex, date of diagnosis, stage, histology, morphology, vital status and date last seen. Histological groupings were created according to criteria specified by the WHO International Classification of Diseases for Oncology (ICD-O-3) (Barnes et al., 2005; Ou et al., 2007) and included: keratinizing squamous cell carcinoma (ICD-O histology codes 8070 and 8071), differentiated non-keratinizing carcinoma (ICD-O histology codes 8072 and 8073), undifferentiated non-keratinizing carcinoma (ICD-O histology codes 8020, 8021, and 8082) and ‘carcinoma not otherwise specified’ (ICD-O histology code 8010). 

*Incidence trends*

Incidence trend analyses were conducted using Joinpoint Regression Program version 4.5.0.1. (Kim et al., 2000) to assess trends under a log-linear model and to compute the annual percent change (APC) in age-standardized incidence rates. The aforementioned program utilizes a Monte Carlo permutation method in order to assess number of joinpoints, slope in the trends, as well as the corresponding significance. Sex stratified trends were assessed by registry and morphological subtype. In circumstances where no cases were reported within a given year, a half-case was added to the age strata with the largest population to enable computation on the log-linear scale (Argirion et al., 2019a; Demanelis et al., 2015; Kim et al., 2000). In order to improve comparability between the various Thai registries and SEER, Segi (1960) standardization was applied (Bray et al., 2002; Segi et al., 1960). R-statistical software version 3.3.3 was used to shape the data and generate graphs based on Joinpoint outputs. Due to small sample sizes of sex stratified differentiated and undifferentiated non-keratinizing carcinomas within the Thailand registries, incidence trends for these morphological subtypes were grouped into a single non-keratinizing category. The A/PI race group was selected as a comparison group from SEER. 

*Survival Analysis*

Kaplan Meier curves assessing differences in survival by sex, year of diagnosis (in five-year groupings) and histological subtype were generated for each registry and strata were compared using log-rank tests. Since Thailand does not have data on expected survival by age, sex, calendar year and province, a life table was ascertained from the WHO Global Health Observatory data repository for the years of 2000-2014 to calculate 5-year relative survival among the Thai registries. Five-year relative survival was computed among Asian/Pacific islanders, for the corresponding years, by sex and subtype, using the Kaplan-Meier method in SEER*Stat 8.3.5. 

## Results

Between 1990 and 2014, 1,894 cases of NPC were diagnosed among A/PIs in the United States, 934 in Chiang Mai, 704 in Khon Kaen, 549 in Songkhla and 390 in Lampang. Cases were found to be predominantly male across all the registries, with mean age at diagnosis ranging from 47-53 years. Among A/PI in the US, the non-keratinizing subtype predominated, making up 51% of cases for both males and females. In Chiang Mai, Khon Kaen and Lampang, non-keratinizing nasopharyngeal carcinoma constituted 66-79% of cases, with no significant differences in distribution between genders within each given registry. Interestingly, in Songkhla the majority of cases were found to be of the keratinizing subtype, comprising about 60% of all reported NPCs (Table 1).

At the beginning of the study period, in 1990, US A/PIs had the highest overall NPC incidence, with an expected age standardized rate (EASR) of 4.48 and 1.60 per 100,000 among males and females, respectively. As time progressed, incidence among A/PI men decreased with an APC of -1.9 (95%CI: -2.6, -1.2) and -1.8 (95%CI: -2.8, -0.8) among women. Declines in NPC incidence were similarly observed among Chiang Mai males, at an APC of -2.2 (95%CI: -3.4, -0.9). Khon Kaen and Lampang presented with suggested increases in nasopharyngeal cancer incidence among both sexes, largely driven by increases in the non-keratinizing subtype. Accounting for these changes in trends, in 2014, US A/PI males remained the highest incident group of NPC (EASR: 2.81 per 100,000), while Lampang emerged as having the highest rates among women (EASR: 1.28 per 100,000) (Figure 1). 

Assessing trends by the presence of keratinization revealed consistent decreases in the keratinizing NPC sub-type; significant decreases were observed among Chiang Mai men between 1996 and 2014 (APC: -13.0 [95%CI: -16.2, -9.6]), Songkhla females (APC: -4.0 [95%CI: -7.4, -0.5]) and males between 2006 and 2014 (APC: -15.5 [95%CI: -25.0, -4.7]), as well as A/PI females (APC: -5.1 [95%CI: -6,7, -3.4]) and males (APC: -4.8 [95%CI: -5.9, -3.7]). Conversely, non-keratinizing NPC appeared to be increasing, particularly among men, across all the registries except Chiang Mai and the US. This observation was found to have the greatest significant impact in Songkhla males, with an APC of 4.3 (95%CI: 1.8, 6.9) (Figure 1).

Sex stratified Kaplan-Meier (KM) plots demonstrated significantly improved survival among females in Songkhla, Khon Kaen and Chiang Mai (logrank p: 0.01, 0.002 and 0.001, respectively) but no differences across the other registries. 

**Figure 2:**
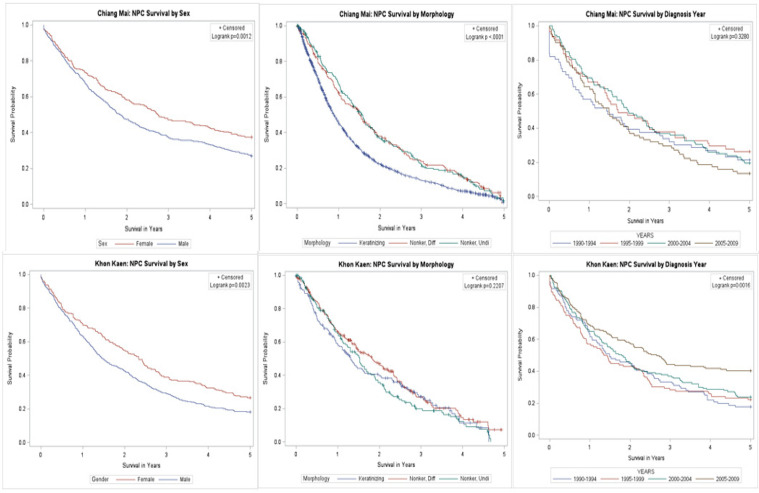
KM Survival Curves of NPC Survival by Sex, Morphology and Year of Diagnosis

The keratinizing squamous cell carcinoma subtype was found to elicit the worst survival, with significant differences noted in Chiang Mai (logrank p: <0.0001), Songkhla (logrank p: <0.0001) and US A/PIs (logrank p: 0.0004). While the nonkeratinizing differentiated subtype appeared to provide the greatest survival advantage in Songkhla, undifferentiated NPC was associated with improved survival among A/PIs. Khon Kaen was the only registry to demonstrate improved survival with later years of diagnosis (logrank p: 0.002) (Figure 2). Five-year relative survival estimates, between the years of 2000 and 2014, closely mirrored the KM results. Although US A/PI had the highest 5-year relative survival estimates, Chiang Mai had the best 5-year relative survival among the Thai registries, while Lampang had the worst survival (Table 2). 

**Table 1:** Distribution of Cases by Gender and Registry (1990-2014)

	USA-Asian/Pacific Islander		Chiang Mai		Khon Kaen		Songkhla		Lampang	
	Male	Female	Male	Female	Male	Female	Male	Female	Male	Female
Mean age (SD), y	52.87 (13.83)	51.76 (15.31)	52.76 (14.11)	51.97 (14.21)	53.02 (14.78)	51.50 (14.46)	51.08 (14.53)	47.17 (16.29)	53.26 (14.62)	51.97 (16.75)
Number of Cases, N (%)	1337 (70.59)	557 (29.41)	650 (69.59)	284 (30.41)	491 (69.74)	213 (30.26)	399 (72.68)	150 (27.32)	261 (66.92)	129 (33.08)
Cell Type, N (%)										
Keratinizing	320 (23.93)	122 (21.90)	178 (27.38)	59 (20.77)	139 (28.31)	57 (26.76)	240 (60.15)	86 (57.33)	72 (27.59)	37 (28.68)
Non-Keratinizing	686 (51.31)	284 (50.99)	466 (71.69)	223 (78.52)	344 (70.06)	150 (70.42)	156 (39.10)	60 (40.00)	178 (68.20)	85 (65.89)
Differentiated	284 (41.40)	130 (45.77)	231 (49.57)	81 (36.32)	199 (57.85)	89 (59.33)	37 (23.72)	14 (23.33)	101 (56.74)	47 (55.29)
Undifferentiated	402 (58.60)	154 (54.23)	235 (50.43)	142 (63.68)	145 (42.15)	61 (40.67)	119 (76.28)	46 (76.67)	77 (43.26)	38 (44.71)
NOS	331 (24.76)	151 (27.11)	6 (0.92)	2 (0.70)	8 (1.63)	6 (2.82)	3 (0.75)	4 (2.67)	11 (4.21)	7 (5.43)

NOS, not otherwise specified

**Table 2:** 5-Year Relative Survival by Registry, Sex and Histology 2000-2014

Number of Cases, N (%)	USA- Asian/Pacific Islander		Chiang Mai		Khon Kaen		Songkhla		Lampang	
	Male	Female	Male	Female	Male	Female	Male	Female	Male	Female
	824	338	395	188	341	151	270	97	181	90
Cell Type, N (%)										
Overall Nasopharyngeal	64.22 (60.46, 67.72)	69.14 (63.27, 74.27)	29.75 (19.84, 39.67)	39.90 (22.05, 57.74)	20.59 (10.89, 30.29)	18.80 (10.76, 26.84)	23.94 (13.99, 33.88)	27.32 (15.08, 39.57)	15.74 (2.41, 29.07)	15.91 (0.00, 32.10)
Keratinizing	54.28 (45.44, 62.28)	52.72 (38.28, 65.26)	29.67 (11.66, 47.67)	36.02 (9.28, 62.76)	13.12 (1.57, 24.67)	25.89 (14.93, 36.85)	31.87 (17.23, 46.51)	33.20 (17.54, 48.86)	18.24 (0.00, 40.73)	13.76 (0.00, 27.58)
Non-Keratinizing	70.79 (65.83, 75.16)	73.54 (65.57, 79.95)	30.76 (20.74, 40.79)	40.46 (27.51, 53.41)	22.54 (15.04, 30.05)	20.88 (11.64, 30.12)	21.69 (10.85, 32.53)	27.87 (10.27, 45.46)	9.70 (2.03, 17.36)	15.48 (1.61, 29.34)
Differentiated	69.37 (61.32, 76.08)	70.53 (58.11, 79.88)	27.35 (11.52, 43.18)	43.95 (23.17, 64.83)	19.42 (8.03, 30.81)	20.82 (7.38, 34.27)	17.40 (0.00, 37.59)	3.36 (0.00, 12.01)	9.79 (0.47, 19.12)	9.05 (0.00, 25.94)
Undifferentiated	71.66 (65.14, 77.18)	76.28 (65.19, 84.26)	34.18 (20.06, 48.30)	37.66 (19.10, 56.23)	25.89 (14.93, 36.85)	20.92 (6.68, 35.16)	24.91 (11.42, 38.40)	41.23 (17.16, 65.30)	9.57 (0.00, 24.75)	22.44 (0.00, 47.11)

## Discussion

While numerous studies on nasopharyngeal carcinoma have been conducted within Chinese populations (Chan et al., 2000; Hu et al., 1991; King and Haenszel, 1973; Yu et al., 1981; Zou et al., 1994), this is the first paper, to our knowledge, to assess temporal trends in NPC incidence and mortality across Thailand in comparison to Asian/Pacific Islanders in the United States. As an endemic country for NPC undergoing rapid economic development, it is important to evaluate the role of both preventative risk factors on incidence, as well as changes in healthcare and treatment protocols on survival among NPC patients in Thailand. In this study, we found overall NPC incidence to be decreasing among US A/PIs of both sexes, as well as Chiang Mai males; nevertheless, the remainder of our study sites displayed stagnant NPC incidence. We additionally found distinct survival differences by sex, registry, and subtype, reflective of the variability of population dynamics, cultural norms and access to care.

Use of combustible tobacco has repeatedly been shown to influence the risk of cancer development across a number of head and neck cancer sites, including the nasopharynx. A recent study in Singaporeans found that current smokers exhibited a four-fold increase in NPC development when compared to never smokers, while ever-smokers exhibited a 2-fold increase (Yong et al., 2017). Similar findings have been reported in Thailand (Ekburanawat et al., 2010; Fachiroh et al., 2012), China (Ji et al., 2011; Yuan et al., 2000), Taiwan (Cheng et al., 1999), the Philippines (West et al., 1993) and the United States (Chow et al., 1993; Mabuchi et al., 1985; Nam et al., 1992; Vaughan et al., 1996). A meta-analysis of the current literature demonstrated that the risks of combustible tobacco use on NPC were particularly relevant to keratinizing squamous cell carcinoma (Yong et al., 2017). Largely due to cultural norms, male smoking rates are much higher than those among female across Thailand; nevertheless, Thailand does display geographic variability in smoking, with the south having the highest smoking prevalence (Benjakul, 2012). Initiated in 1991, Thailand was among the first countries in Asia to implement strict tobacco control policies, including bans on advertisements and smoking in certain public places, health warnings and taxation (Chantornvong et al., 2000; Chantornvong and McCargo, 2001; Haman, 2003; Vateesatokit et al., 2000). According to the Tobacco Control Research and Knowledge Center, smoking prevalence has decreased across Thailand, but smoking behaviors remain highest in the south (decreasing from 60.9% in 1994, to 49.9% in 2007) (2008; Chang et al., 2018). The greater proportion of male smokers likely contributes to the higher incidence of keratinizing squamous cell carcinoma among males observed across all study sites. Songkhla (located in southern Thailand) was the only registry to exhibit higher keratinizing rather than non-keratinizing NPC incidence, likely due to the high smoking rates within the region. 

Keratinizing squamous cell carcinoma typically predominates in non-endemic regions such as the United States (Wang et al., 2013). Nevertheless, previous studies have shown that Asian Americans continue to exhibit higher rates of NPC, particularly the non-keratinizing subtype, when compared to other race groups (Argirion et al., 2019b; Wang et al., 2013). In this study, we found US A/PIs to have the highest age standardized rate of NPC, when compared to the Thai registries. Decreases in incidence over the time period resulted in Lampang females superseding US A/PI females, US A/PI males remained the highest incident group. Although, this study validated previous observations of a predominance of non-keratinizing NPC among this population, the decreased incidence observed within overall NPC is largely attributable to decreases in keratinizing squamous cell carcinoma, while the non-keratinizing subtype remained stagnant. 

While non-keratinizing NPC is believed to present with a more aggressive behavior and a higher risk of distant metastasis, keratinizing squamous cell carcinoma has been reported to be less responsive to treatment (Paiar et al., 2012). Historically, radiotherapy has been the cornerstone of treatment across NPC subtypes. In the minority of patients diagnosed in early stages, NPC has relatively high sensitivity to radiation, but this treatment has been shown to fail among patients with locally advanced disease (Paiar et al., 2012). More recently, concurrent chemo-radiation treatment has been shown to improve survival among non-keratinizing locally advanced patients, particularly those with undifferentiated tumors (Paiar et al., 2012; Perri et al., 2011). Chiang Mai was the first registry location in Thailand to implement the use of chemoradiation in NPC treatment, beginning enrollment in a clinical trial in 1989 and adapting the treatment into practice in 1996 (Chua et al., 1998). Shortly upon completion of the phase 3 randomized Intergroup 0099 Study in 1995 (Al-Sarraf et al., 1998), the US adopted chemoradiation as the primary treatment for advanced stage NPC; Khon Kaen, Lampang and Songkhla similarly implemented the new treatment guidelines soon after the US (Pornthep Kasemsiri, 2015). Coinciding with the literature, our study found significantly worse survival among those diagnosed with keratinizing squamous cell carcinoma in Chiang Mai, Songkhla, and US A/PI. Undifferentiated non-keratinizing NPC appeared to be associated with improved survival in Chiang Mai, Lampang and the US, likely due in part to tumor treatment response, and in part to the earlier age of onset for this cancer subtype (data not shown) (Leu et al., 2014). Five-year relative survival results indicated that US A/PIs have the best survival, regardless of cancer subtype. Among the Thai registries, Chiang Mai proved to provide the best survival rates, likely due to the early adaptation of chemoradiation.

Although the early adaptation of improved treatment modalities may improve patient survival, equal access to care remains a problem in parts of Thailand. Northern Thailand is unique in that it encapsulates the ‘hill tribe’ population. These individuals reside in the mountain slopes and are divided into six primary groups: Akha, Lahu, Hmong, Lisu, Yao and Karen. In addition to having different cultural practices, the hill tribes have unique languages, as well as beliefs that influence their health care practices (Apidechkul et al., 2016b; Besaggio et al., 2007). Originating largely from Southern China (a high incidence region for NPC), the Akha are the largest tribe, comprised of an estimated 70,000 individuals (Apidechkul et al., 2016b). Historically migrant populations, many hill tribe residents did not have legal identification and were not considered Thai citizens until recently. A 2014 report by the Mae Fah Luang District Office estimated that 89% of hill tribe residents had access to health care under the Thai Universal Coverage System, which was introduced in 2000 (Apidechkul et al., 2016a; Chiang Rai, 2014). In 2011, the National Health Examination Survey Office reported that smoking rates among Ahka young adults was higher than that of Thai young adults (28% and 19.9%, respectively) (2011). While exhibiting various high risk behaviors, previous studies have demonstrated that factors such as language barrier/illiteracy rates, lack of transportation, financial restrictions and reliance on traditional medical practices are all factors that contribute to health disparities within hill tribe populations (Apidechkul et al., 2016a; Apidechkul et al., 2016b; Pitchayakan, 1982; Rittirong et al., 2014). These various obstacles consequently impact utilization of both treatment and preventative care. Unlike trends observed in Khon Kaen and Songkhla, the Chiang Mai and Lampang registries appear to have worsening survival with successive years of diagnosis, particularly corresponding to the initiation of universal healthcare. Due to limitations in access to care, these trends may be attributable to an increase of hill tribe patients, who would likely be diagnosed at later stages and be less willing and able to comply with modern treatment protocols. 

Differences in data collection methods and population composition pose challenges to comparability between registries. Although Thailand’s cancer registries have been verified have high quality data (Argirion et al., 2019a; Suwanrungruang et al., 2011), data collection methods vary from passive, to active, to a combination of the two (Chitapanarux, 2018; Pongnikorn, 2018; Sriplung, 2018; Wiangnon, 2018). Additionally, while population-based cancer registries allow results to be generalized to nearby provinces, it is difficult to quantify the precise number of unreported cancer cases, particularly within rural areas. Finally, changes to histology practices, such as the introduction of immunohistochemistry staining, may influence subsite specific classification, leading to misclassification bias. This effect is likely observed in the Chiang Mai and Songkhla registries, where the apparent trends between keratinizing and non-keratinizing NPC incidence invert around 1996 and 2006, respectively. 

In conclusion, albeit not being an endemic country for NPC, US A/PIs appear to have comparable incidence rates to those observed in the endemic Thai population—even exceeding the cancer burden observed among Thai males. This study also demonstrated the role of tobacco control on keratinizing NPC incidence, as well as the survival benefit associated with the introduction of chemoradiation in non-keratinizing NPC treatment. Finally, it puts into perspective the challenges associated with access to care in rural populations, irrespective of the introduction of universal health care. In order to address this and other potential health disparities in these communities, local health professionals should be encouraged to develop culturally appropriate health promotion and educational programs in order to aid in curbing high-risk behaviors and improving access and acceptance of care. 
